# Perception of the food environment by Brazilian slum residents: a qualitative study

**DOI:** 10.1590/0102-311XPT128423

**Published:** 2024-03-22

**Authors:** Luana Lara Rocha, Amélia Augusta de Lima Friche, Mariana Zogbi Jardim, Paulo César Pereira de Castro, Emanuelly Porto Oliveira, Letícia de Oliveira Cardoso, Larissa Loures Mendes

**Affiliations:** 1 Universidade Federal de Minas Gerais, Belo Horizonte, Brasil.; 2 Universidade Federal do Rio de Janeiro, Rio de Janeiro, Brasil.; 3 Escola Nacional de Saúde Pública Sergio Arouca, Fundação Oswaldo Cruz, Rio de Janeiro, Brasil.

**Keywords:** Perception, Access to Healthy Foods, Slums, Percepção, Acesso a Alimentos Saudáveis, Favelas, Percepción, Acceso a Alimentos Saludables, Favelas

## Abstract

Food availability in the territory can influence food consumption by the population. However, it is important to understand how people perceive their food environment to see how food availability affects consumption in different contexts. This study aimed to assess the perception of the food environment by Brazilian slum residents in their neighborhoods. This is a qualitative study, with online focus groups guided by a script in order to gather collective discourses about access to food in Brazilian slums. The invitation to participate in this study was made through social media, and community leaders and nongovernmental organizations with actions in slums were contacted using the snowball sampling technique. Grounded theory analysis was applied with the technique of thematic networks. Access to food for slum residents involves lack of resources and essential elements for an adequate and healthy diet, such as lack of information about food, low income, and low availability of stores that sell healthy food at affordable prices. Public programs and policies are required to encourage the expansion of food and nutritional security resources, such as vegetable gardens and markets, to increase the supply and sell healthy food at affordable prices in slums. Actions are also required to address the complexity of obstacles faced by slum residents in the access to healthy foods.

## Resumo

A disponibilidade de venda de alimentos no território pode influenciar no consumo alimentar da população. Entretanto, é importante compreender como as pessoas percebem o seu ambiente alimentar para se entender como essa disponibilidade afeta seu consumo em contextos distintos. O objetivo foi avaliar a percepção dos moradores de favelas brasileiras sobre o ambiente alimentar em suas vizinhanças. Estudo qualitativo, em que foram realizados grupos focais *online* guiados por um roteiro para reunir discursos coletivos sobre o acesso aos alimentos em favelas brasileiras. O convite ocorreu por meio das redes sociais e do contato com líderes comunitários e organizações não governamentais que atuam em favelas, por meio da técnica de amostragem “bola de neve”. Para a análise, foi utilizada a abordagem *grounded theory* (teoria fundamentada) e, como técnica, foi empregada a análise de redes temáticas. O acesso aos alimentos por moradores de favelas é permeado pela falta de recursos e elementos fundamentais para uma alimentação adequada e saudável, como: a falta de informação sobre alimentação; a renda insuficiente; e a baixa disponibilidade de estabelecimentos que comercializam alimentos saudáveis a preços acessíveis. São necessários programas e políticas públicas que incentivem a ampliação de equipamentos de segurança alimentar e nutricional, como hortas e feiras, e que aumentem a oferta de alimentos saudáveis com valores baixos nas favelas. Também são necessárias ações que abordem a complexidade das barreiras enfrentadas por moradores de favelas para ter acesso aos alimentos saudáveis.

## Resumen

La disponibilidad de venta de alimentos en el territorio puede influir en el consumo alimentario de la población. Sin embargo, es importante comprender la manera en que las personas perciben su entorno alimentario para comprender cómo esta disponibilidad afecta su consumo en diferentes contextos. El objetivo fue evaluar la percepción de los residentes de favelas brasileñas sobre el entorno alimentario en sus vecindarios. Estudio cualitativo, en el que se formaron grupos focales en línea orientados por un guion con el objetivo de reunir discursos colectivos sobre el acceso a los alimentos en favelas brasileñas. La invitación se dio por medio de las redes sociales y mediante el contacto con líderes comunitarios y organizaciones no gubernamentales que trabajan en favelas, utilizando la técnica de muestreo “bola de nieve”. Para el análisis, se utilizó el enfoque de la *grounded theory* (teoría fundamentada) y, como técnica, se empleó el análisis de redes temáticas. El acceso a los alimentos de los habitantes de las favelas está permeado por la falta de recursos y elementos fundamentales para una alimentación adecuada y sana, tales como: la falta de información sobre la alimentación, los bajos ingresos y la poca disponibilidad de establecimientos que vendan alimentos sanos a precios asequibles. Se necesitan programas y políticas públicas para fomentar la ampliación de equipos de seguridad alimentaria y nutricional, como huertas y mercadillos, que aumenten la oferta y vendan alimentos sanos a precios asequibles en las favelas. También se necesitan acciones para abordar la complejidad de las barreras que enfrentan los residentes de las favelas para acceder a alimentos sanos.

Este é um artigo publicado em acesso aberto (Open Access) sob a licença Creative Commons Attribution, que permite uso, distribuição e reprodução em qualquer meio, sem restrições, desde que o trabalho original seja corretamente citado.

## Introdução

O acesso permanente e regular aos alimentos saudáveis, em quantidade e qualidade suficientes, é um dever do Estado garantido pelo Direito Humano à Alimentação Adequada (DHAA) [1]. Conforme orientado no *Guia Alimentar para a População Brasileira* [2], uma alimentação saudável deve ser baseada em alimentos *in natura* e minimamente processados, evitando o consumo de alimentos ultraprocessados. No entanto, o acesso aos alimentos não é igualitário no Brasil. Estudos revelam que pessoas em áreas socioeconomicamente vulneráveis percebem menor proximidade de varejistas de alimentos [3,4]. Nessas áreas, o acesso aos alimentos saudáveis é mais desafiador, causando impacto em seu consumo [5,6,7]. Lopes et al. [8], ao avaliarem as associações entre a segregação residencial econômica e a prevalência do consumo de marcadores alimentares saudáveis e não saudáveis por adultos residentes em uma metrópole brasileira, evidenciaram que moradores de bairros altamente segregados têm menor consumo regular de frutas e legumes em comparação com moradores de bairros mais ricos.

Dessa forma, os estudos disponíveis avaliam e caracterizam o ambiente alimentar formal em áreas vulneráveis, relacionando-o à renda e às condições socioeconômicas locais. Isso sugere que, devido à vulnerabilidade social, as favelas têm um ambiente alimentar distinto da cidade formal – caracterizada por investimentos concentrados, infraestrutura sofisticada e presença estatal marcante –, sendo uma vizinhança que dificulta o consumo de alimentos saudáveis. Essa discrepância demanda estudos específicos sobre o ambiente das favelas, definidas como ocupações irregulares para fins de habitação em áreas urbanas [9].

O conceito de ambiente alimentar compreende a interface entre o consumidor e o sistema alimentar, abrangendo disponibilidade, acessibilidade, conveniência, promoção, qualidade e sustentabilidade de alimentos em espaços formais e informais, influenciado pelo ambiente sociocultural, político e pelos ecossistemas [10]. Avaliar como a população que reside em favelas percebe o seu ambiente alimentar auxilia-nos na compreensão da relação entre o acesso físico e econômico aos alimentos e o seu consumo. Alber et al. [11] realizaram uma pesquisa na Filadélfia (Estados Unidos) para observar a associação da percepção de adultos residentes em bairros de baixo e alto nível socioeconômico do ambiente alimentar com o acesso físico, o preço e a qualidade de frutas e vegetais. Os autores encontraram que a percepção do acesso físico e da qualidade de frutas e hortaliças na vizinhança foram associadas positivamente com o consumo desses alimentos.

A utilização de instrumentos sobre a percepção do ambiente alimentar ainda não foi realizada no Brasil, devido à necessidade de validação para o contexto brasileiro [12]. Para preencher essa lacuna na literatura e avançar nas investigações sobre o ambiente alimentar de favelas, este estudo adotou uma abordagem qualitativa para explorar a percepção dos moradores de favelas brasileiras sobre o ambiente alimentar, ao avaliar a dimensão do acesso aos alimentos em suas vizinhanças. Compreender a percepção dos moradores de favelas sobre o seu ambiente alimentar é crucial para se entender como o acesso físico e financeiro aos alimentos podem afetar seu consumo em contextos distintos. Ademais, as características dos bairros podem afetar a saúde e contribuir para as desigualdades sociais na saúde e no acesso aos alimentos [13].

## Métodos

### Desenho do estudo

Estudo qualitativo realizado com moradores de favelas brasileiras com o objetivo de identificar a percepção sobre o ambiente alimentar nesses locais. A hipótese do estudo é que moradores de favelas têm acesso dificultado aos alimentos *in natura* e minimamente processados, devido às condições socioeconômicas e ambientais desses locais. Para a condução do estudo, foram realizados grupos focais com o objetivo de reunir discursos coletivos sobre o acesso físico e financeiro aos alimentos em favelas brasileiras.

Os grupos focais foram realizados de forma remota em dois diferentes dias. O formato *online* foi escolhido para permitir a participação de moradores de favelas de diversas regiões do Brasil de forma conjunta. Além da facilidade de acesso aos recursos com ampla cobertura geográfica, esse formato fomenta o avanço das tecnologias, arquivamento seguro de dados, baixo custo, minimização do efeito da influência de grupos e possível anonimato [14]. A fim de minimizar a limitação inerente à técnica, relacionada à possibilidade de interferência dos moderadores quanto aos juízos de valores, a condução dos grupos foi realizada por um pesquisador experiente e treinado em técnicas qualitativas de pesquisa e análise de dados, com auxílio de dois observadores treinados.

Este estudo está relatado de acordo com *Standards for Reporting Qualitative Research* (SRQR; Padrões para Relatórios de Pesquisa Qualitativa) [15] para relatórios qualitativos de pesquisa.

## Participantes

Os participantes elegíveis para o estudo foram pessoas residentes em favelas brasileiras maiores de 18 anos. O convite ocorreu por meio das redes sociais e do contato com líderes comunitários e organizações não governamentais que atuam em favelas, por meio da técnica de amostragem "bola de neve". A amostra por bola de neve é uma técnica não probabilística, na qual os indivíduos selecionados convidam novos participantes da sua rede de amigos e conhecidos.

Os interessados em participar do grupo focal com o tema "ambiente alimentar de comunidades, vilas e favelas brasileiras" preenchiam um formulário *online* com as informações para a caracterização sociodemográfica do participante e escolhiam um dia para a sua participação remota. A definição de favela realizada pelo Instituto Brasileiro de Geografia e Estatística (IBGE) estava no formulário de inscrição para o grupo focal; dessa forma, os participantes que se inscreveram consideraram que residiam nessas áreas. Os participantes receberam lembretes do compromisso com o grupo focal no dia anterior, por aplicativo de mensagens, minimizando possíveis perdas. Como contrapartida e também com o intuito de melhorar a adesão aos grupos, foram enviados certificados de participação.

## Coleta de dados e estratégia analítica

Foram conduzidos dois grupos focais com duração aproximada de 120 minutos, no mês de novembro de 2022, pela plataforma Zoom (https://zoom.us/pt). Antes de iniciar a entrevista, houve uma apresentação do moderador, esclarecendo a intenção do trabalho e a importância da gravação do encontro, ratificando que não havia resposta certa ou errada.

Para guiar os grupos, foi utilizado um roteiro (Material Suplementar – Quadro S1; https://cadernos.ensp.fiocruz.br/static//arquivo/suppl-e00128423_7789.pdf) baseado nos blocos de perguntas sobre a compra de alimentos e refeições fora de casa, da versão adaptada e traduzida para o Brasil do instrumento *Perceived Nutrition Environment Measures Survey* (NEMS-P; Pesquisa de Medidas Percebidas do Ambiente Nutricional) [12]. O roteiro consistia em questões abertas centrais e secundárias, que convergiam para o objetivo da pesquisa (Quadro 1), com poucas perguntas a fim de permitir flexibilidade com registro dos temas abordados e evitar respostas conclusivas que não estimulassem a discussão dos participantes [16,17].

Os grupos focais foram gravados e passaram por uma transcrição literal do áudio, identificando quais participantes contribuíram com cada fala. A partir das gravações, foram realizadas facilitações visuais para ilustrar de forma gráfica as principais falas dos participantes por um profissional experiente nesse tipo de técnica (Material Suplementar – Figuras S1 e S2; https://cadernos.ensp.fiocruz.br/static//arquivo/suppl-e00128423_7789.pdf).

Para a análise, foi utilizada a abordagem *grounded theory* (teoria fundamentada) e, como técnica, foi empregada a análise de redes temáticas (ART). Tendo em vista a natureza descritiva da pesquisa e dos dados, fez-se necessária uma análise exploratória, embasada em uma abordagem teórica fundamentada nos dados. Tal perspectiva tem como característica central a possibilidade de os dados coletados informarem as teorias e cenários sociais, ao contrário de preestabelecer o teste de hipóteses como ensaio central [18].

A ART é uma técnica de investigação que resume e sistematiza os principais temas e padrões de dados textuais e explora a compreensão de uma questão ou um problema e os significados e sentidos de ideias de indivíduos em determinado contexto. Essa técnica torna possível uma sistematização dos dados textuais, permite a divulgação de cada etapa do processo analítico e ajuda na organização da análise e da apresentação dos dados [19].

**Quadro 1**

Temas centrais do roteiro utilizado para guiar os grupos focais.

| ROTEIRO | |
|---|---|
| **TEMA CENTRAL** | **DIMENSÕES AVALIADAS** |
| Conceitos sobre a alimentação | O que é a alimentação saudável e a não saudável<br>Informação sobre alimentos e alimentação<br>Percepção sobre a alimentação de cada pessoa<br>Maiores dificuldades para se ter uma alimentação saudável |
| Ambiente alimentar domiciliar | Alimentos disponíveis nos domicílios |
| Compra e doação de alimentos | Locais onde são comprados os alimentos<br>Percepção sobre o acesso aos alimentos saudáveis na vizinhança e nos locais de compra<br>Presença de propagandas e promoções<br>Utilização de serviços de entrega de alimentos<br>Costume de realizar refeições fora de casa<br>Recebimento de doações |
| Produção de alimentos | Se a pessoa produzia alimentos para consumo próprio<br>Presença de hortas comunitárias ou vizinhos com hortas que vendiam ou doavam alimentos |

A primeira etapa da ART é transformar os dados de texto em segmentos significativos, utilizando um quadro de codificação. Neste trabalho, foi utilizada abordagem teórica fundamentada nos dados, permitindo que o texto mostrasse quais são as questões mais importantes ou recorrentes para a criação da moldura de codificação [19]. No processo de codificação, os códigos para dissecar os dados em segmentos de texto foram aplicados. Os segmentos de texto significativos foram utilizados como unidade de análise [20].

O processo de codificação e análise dos dados adotado no estudo foi baseado no paradigma interpretativo da pesquisa qualitativa [21], aproveitando a experiência do pesquisador que moderou os grupos focais. Nesse sentido, o processo de análise dos dados inicia-se no momento da interlocução com os entrevistados, momento chave para o desenvolvimento de sensibilidade e consenso sobre os contextos, situações e, em última instância, fenômenos descritos. Essa prática é fundamental para o momento da codificação dos dados, pois é no processo dialógico e reflexivo entre entrevistado e entrevistador que os fenômenos se tornam estáveis e, em alguma medida, tornam-se consenso entre as partes [22].

O passo seguinte foi identificar temas a partir dos segmentos de texto codificados. Após reler os segmentos de texto em cada código e extrair os temas comuns ou significativos, os códigos foram refinados no software ATLAS.ti (http://atlasti.com/), de análise de dados qualitativos. Depois de extrair os temas, os subtemas suficientemente específicos foram refinados para não serem repetitivos e suficientemente amplos para unir conjuntos de ideias contidas em muitos segmentos de texto. Os códigos derivados do texto foram utilizados como temas básicos dos grupos focais. Os temas básicos foram alocados em grupos coerentes, que se tornaram redes temáticas.

## Aspectos éticos

Este estudo foi aprovado pelo Comitê de Ética da Universidade Federal de Minas Gerais (CAAE: 54588221.7.0000.5149 e 48190221.2.0000.5149). Todos os participantes leram o Termo de Consentimento Livre e Esclarecido (TCLE) e autorizaram, por meio de um formulário digital, a participação e utilização das falas.

## Resultados

Dos 27 moradores de favelas inscritos, dez participaram efetivamente dos grupos, sendo cinco do Grupo 1 e cinco do Grupo 2. A Tabela 1 apresenta a caracterização dos participantes dos grupos focais. A maioria dos participantes era do sexo feminino (80%), com idades entre 18 e 30 anos (60%), das raças/cores preta (30%), parda (30%) e branca (30%). Esses moradores residiam em favelas localizadas, em sua maioria, na Região Sudeste do Brasil (60%). A caracterização dos moradores inscritos que não participaram das oficinas está no Material Suplementar (Tabela S1; https://cadernos.ensp.fiocruz.br/static//arquivo/suppl-e00128423_7789.pdf).

A partir da análise dos discursos nos grupos focais, foi construída a rede temática, conforme apresentado na Figura 1. O principal achado da análise revelou que o acesso dos moradores de favelas aos alimentos é influenciado pela falta de recursos e elementos fundamentais para uma alimentação saudável, incluindo: informações sobre alimentos e alimentação; renda; e estabelecimentos que disponibilizam alimentos saudáveis a preços acessíveis.

Quando questionados sobre a primeira ideia que lhes ocorre ao pensar em alimentação saudável, os participantes abordaram temas relacionados ao direito ao acesso a alimentos, aspectos socioculturais, saúde física e corporal, bem como características dos alimentos saudáveis (Quadro 2; códigos F1 a F5). A ênfase recaiu, principalmente, sobre as características nutricionais dos alimentos saudáveis (Quadro 2; códigos F2, F4 e F5). Ao definirem a alimentação saudável, os participantes citaram alimentos orgânicos, vegetais, carnes, arroz e feijão como exemplos. A dimensão biológica dos alimentos também foi destacada, com ênfase em conteúdos como teor de sal, açúcar, micronutrientes, proteínas e carboidratos nos alimentos (Quadro 2; códigos F4 e F5). Os participantes também associaram a alimentação saudável à acessibilidade física e econômica. Foi discutido que, antes de abordar as características dos alimentos, é crucial que eles sejam acessíveis e estejam alinhados com as características culturais da população (Quadro 2; códigos F3 e F9).

Quando indagados sobre o que caracteriza uma alimentação não saudável, os participantes mencionaram a dimensão biológica dos alimentos e forneceram exemplos de alimentos considerados não saudáveis, com massas e alimentos ultraprocessados sendo citados com maior frequência (Quadro 2; códigos F6 e F7).

**Tabela 1**

Caraterização dos moradores de favelas brasileiras que participaram dos grupos focais (n = 10).

| Variáveis | n | % |
|---|---|---|
| Sexo | | |
| Feminino | 8 | 80 |
| Masculino | 2 | 20 |
| Idade (anos) | | |
| 18-30 | 6 | 60 |
| 30-50 | 3 | 30 |
| > 50 | 1 | 10 |
| Raça/Cor | | |
| Preta | 3 | 30 |
| Parda | 3 | 30 |
| Branca | 3 | 30 |
| Indígena | 1 | 10 |
| Região do Brasil | | |
| Norte | 2 | 20 |
| Nordeste | 1 | 10 |
| Sul | 1 | 10 |
| Sudeste | 6 | 60 |
| Centro-oeste | - | - |

**Figura 1**

Análise de redes temáticas da percepção de moradores de favelas brasileiras em relação ao acesso aos alimentos.

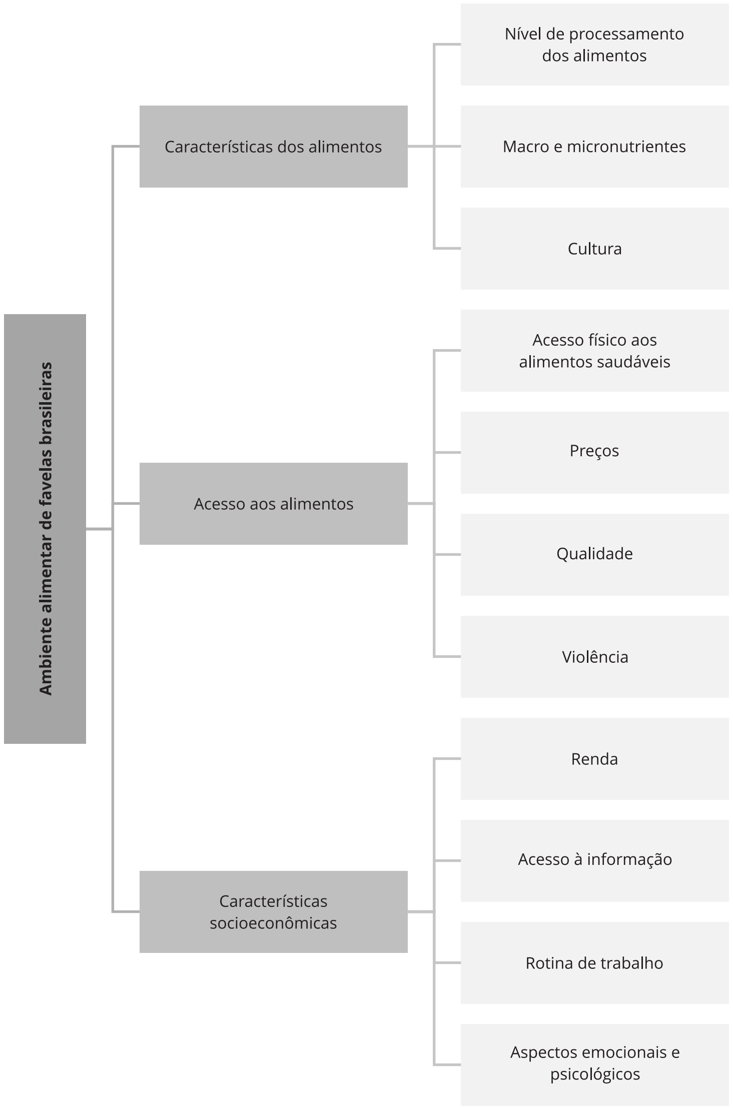

Outra dimensão mencionada pelos participantes foi a razão pela qual não conseguem manter uma alimentação saudável. As principais justificativas foram a escassez de recursos financeiros, a falta de informações e a falta de tempo para preparar refeições (Quadro 2; códigos F8 a F10).

Entretanto, no geral, os participantes consideraram que tinham uma alimentação saudável. Relataram que buscam equilíbrio em suas refeições, consomem vegetais e evitam alimentos ultraprocessados. Mas reconheceram que ainda havia espaço para melhorias. Embora a maioria tenha relatado o consumo diário de verduras, legumes, frutas, carnes, arroz e feijão, alguns participantes mencionaram que não consomem esses alimentos com frequência devido aos preços elevados. Os participantes indicaram que aumentariam o consumo e a variedade de frutas e alimentos orgânicos, peixes, castanhas e sementes se tivessem condições financeiras. Também mencionaram que ter tempo disponível para diversificar os tipos de alimentos e preparar refeições com maior qualidade era uma aspiração

**Quadro 2**

Síntese dos principais resultados agrupados por dimensões do ambiente alimentar e do acesso aos alimentos.

| SÍNTESE DOS RESULTADOS | | |
|---|---|---|
| **DIMENSÕES** | **CÓDIGO** | **RESPOSTA** |
| O que é a alimentação saudável e a não saudável | F1 | *"Não é o café farto, assim, dentro das possibilidades da gente né, um pãozinho, um biscoito, uma fruta, um cafezinho, depois um almoço, um lanchinho entre intermediário o café da manhã e o almoço né?"* (Grupo 1) |
| | F2 | *"Tanto a questão da qualidade, da qualidade né, e para o nosso corpo funcionar. Então, quando eu penso na alimentação que tem essa base né da quantidade e da qualidade pra gente poder fazer o nosso corpo e nossa mente funcionar"* (Grupo 2) |
| | F3 | *"Não respeitar a diversidade cultural daquele ambiente, por exemplo, se for pra fazer uma cesta básica na região aqui onde eu moro em Sergipe, tem que ter um cuscuz,* [risos], *tem que ter um alimento que é típico, entendeu?"* (Grupo 1) |
| | F4 | *"Bem, os alimentos saudáveis pra mim é as frutas, as verduras, os legumes, né, a gente deixar de ingerir sal, não ingerir muito açúcar, pra quem é diabético de jeito nenhum, né, então assim alimentação saudável é não contar muito carboidrato, isso aí, naturais, sem conservante, é o que a gente tem sempre levado à população, a diminuir os conservantes e sal, e investir mais na alimentação sem agrotóxico também, né? Mais, é... legumes e frutas"* (Grupo 2) |
| | F5 | *"Eu entendo que é ter acesso aos alimentos básicos, né, aquela famosa prato coloridinho, aquela pirâmide alimentar, em que tem o arroz e feijão, talvez uma fonte de proteína, ter acesso a frutas, legumes e verduras frescas"* (Grupo 2) |
| | F6 | *"Tá, aí eu cito acho que os ultraprocessados como os principais vilões, assim, a gente vê aumentando o consumo desse tipo de alimento nos últimos anos, é, são alimentos com grande quantidade de sal, açúcar e gordura e muitos deles com uma grande concentração de calorias, é... que... todos esses componentes consumidos em excesso tendem a piorar a qualidade da alimentação e trazer... aumentar os riscos né, doenças associadas à alimentação"* (Grupo 2) |
| | F7 | *"Alimentos processados, por exemplo em lugares de favela que é onde eu atuo são muitos alimentos processados com muito carboidrato, muita massa, enfim, muita coisa mais pesada"* (Grupo 1) |
| Informação sobre alimentos e alimentação | F8 | *"Uma é que não sabem fazer as coisas, o conhecimento não chega, assim, entre comer, sei lá, montar um prato com arroz e feijão, aí bota arroz, feijão, macarrão, farofa e batata palha por cima, então não sabe fazer a escolha inteligente"* (Grupo 2) |
| | F9 | *"É que eu vou sempre ir além do que seria mais objetivo porque na prática a gente observa muito isso, tem muita gente que sabe se alimentar, ter esses alimentos variados e conseguir esse acesso às informações do que seria mais saudável, mas tem a impossibilidade de ter esse acesso a esse direito por conta mesmo da falta de recursos a ter essa variação"* (Grupo 1) |
| Percepção sobre a alimentação de cada pessoa | F10 | *"Então, tipo, eu... eu sacio muita a minha fome com a água, porque quando começa a pegar o bicho aí eu vou na garrafinha de água até chegar em casa pra fazer o almoço, mas eu queria que melhorasse muito, fizesse aquela regrinha, comer de 3 em 3 horas. A correria do dia a dia, cê já acorda de manhã, ainda tem que preparar o pessoal pra sair pra trabalhar, não tem... ó, é a correria do dia a dia"* (Grupo 1) |
| | F11 | *"Mas pode melhorar especialmente no jantar no sentido de, é... opções melhores. Então, talvez é meio, eu trabalho de fazer as quentinhas, minhas marmitinhas de fazer no almoço, fazer também pro jantar esse é mais meu desafio ai até o final do ano"* (Grupo 2) |
| | F12 | *"Se as condições econômicas me permitissem, é... castanhas, nozes, adoraria colocar* [risos] *essas, esses alimentos, essas coisinhas, essas coisas diferentes né que a gente geralmente não consegue colocar. Mas, eu queria colocar mais peixe, mais variedade de frutas, sementes, castanhas, eu gostaria de colocar isso mais na minha vida sim"* (Grupo 1) |

(continua)

(Quadro 2; códigos F10 a F12). Além disso, alegaram que questões emocionais e psicológicas, como ansiedade e depressão, afetam seu padrão alimentar, sendo a violência um fator que influencia negativamente essas questões e, consequentemente, o consumo de alimentos (Quadro 2; códigos F13 a F16).

Quanto aos locais de compra de alimentos, as feiras locais, supermercados, mercados locais ou hortifrutis foram os preferidos (Quadro 2; código F17). Alguns participantes mencionaram a dificuldade de comprar em feiras, pois não ocorrem em seus bairros ou estão distantes de suas casas. Além disso, reconheceram que a oferta de alimentos de qualidade é baixa onde vivem, o que os leva a adotar

**Quadro 2 (continuação)**

| SÍNTESE DOS RESULTADOS | | |
|---|---|---|
| **DIMENSÕES** | **CÓDIGO** | **RESPOSTA** |
| Maiores dificuldades para se ter uma alimentação saudável | F13 | "*Olha, eu acho que a minha questão é mais, eu acho que boa parte das pessoas passa por isso, é uma questão mais emocional e que eu carrego desde a infância assim, do fundamental ou ensino médio, ah semana de prova, semana de estudar aquilo de muitos compromissos eu acabo descontando em minha alimentação, acabo incluindo alimentos que eu sei que não são tão legais assim, entendeu? Então, eu acredito que eu poderia melhorar nesse aspecto*" (Grupo 1) |
| | F14 | "*É... a violência também nos territórios de favelas a gente acaba também tendo essa questão da alimentação abalada, muita gente deixa de comer, né, e muita gente se alimenta mais e ruim por conta do acesso, né? Falando agora um pouco de mim, dessa ansiedade que eu tenho, sempre quando eu tenho em dias de tiroteios essas coisas, porque eu tenho uma relação com a comida muito boa, eu gosto muito de comer, então eu como quando eu to triste, eu como quando eu to ansiosa, eu como quando eu to feliz, enfim, eu tenho uma relação com a comida que é muito ampla né? Mas, nesses dias de muita tensão eu fico procurando coisas assim que o paladar se sinta mais satisfeito, né, é... com doces, salgadinhos pacotes e tal, então isso acaba me dando um, talvez, em texturas, enfim, um pouco mais de tranquilidade, mais ou menos né, mas é o que são as minhas válvulas de escape, né, então é isso*" (Grupo 1) |
| | F15 | "*É porque o... como foi dito, a gente tende a comer o que é mais fácil, né? De maneira geral, e... se o ambiente ao nosso redor nos oferece alimentos que não são tão saudáveis a tendência é que a gente consuma mais desses alimentos, né? Então, acho que esse é o maior problema de muitas comunidades assim o ambiente e o que a gente encontra dentro dos comércios e regiões ali dentro, né? Se a pessoa tem uma dificuldade maior pra comprar frutas, legumes e verduras, fazer um grande deslocamento por exemplo, a tendência é que ela coma menos desse tipo de alimento, e isso aí vai fazer com que a qualidade da nossa alimentação vá piorando. É muito fácil a gente cair na armadilha e deixar de consumir, ou consumir com menos frequência as frutas, legumes e verduras*" (Grupo 2) |
| | F16 | "*Eu praticamente fico em jejum né até passar aquele momento ali, até realizar aquele determinado problema, aí eu vou me alimentar. Eu acho que isso é um fator que influencia né no nosso psicológico também, ainda mais hoje em dia que a maioria fala né da doença do século né, então, a gente deveria ter mais cuidado né, relacionado ao alimento, como o ambiente ali pode nos afetar de uma forma tão drástica que acaba até afetando a nossa alimentação*" (Grupo 1) |
| Locais onde são comprados os alimentos | F17 | "*Eu compro mais no supermercado aqui pertinho de casa mesmo, ou nas redes de supermercados né? E... verdura no sacolão, e carne geralmente já to dentro do supermercado geralmente eu procuro já comprar lá dentro nos açougues dos supermercados mesmo*" (Grupo 2) |
| Percepção sobre o acesso aos alimentos saudáveis na vizinhança e nos locais de compra | F18 | "*Porque se no sacolão tiver alguma verdura que não tenha agrotóxico, ai eu vou comprar o que não tem, mas se não tem eu vou levar o que tem lá dentro, entendeu? Então, assim, assim me fez refletir muito que com a pandemia subiu tudo, ficou tudo tão caro, ficava muito triste de ficar sem requeijão, igual eu moro num bairro mais assim de risco elevado, as pessoas meio que não tinha porque houve muito desespero, eu vi as pessoas pegando o que o pessoal desprezada no balde pra jogar fora, elas estavam pegando aquelas verduras, aquelas coisas sabe? Então, quando que a gente tem acesso a gente leva*" (Grupo 2) |
| | F19 | "*Onde eu vivo é mais difícil, a qualidade não é boa e o preço também não é bom. Se eu quiser comprar frutas, legumes e verduras num preço... mais legal e de uma qualidade também, eu tenho que aguardar o varejão, como falam, que acontece dentro do centro comunitário da comunidade, das comunidades da cidade, é... que acontece uma vez por semana, né, ai lá eu consigo comprar legumes e verduras com uma qualidade maior. Se não, eu fico dependendo desses pequenos comércios, é, onde de certa forma tem que fazer um deslocamento maior pra comprar as frutas, legumes e verduras, lá a qualidade não é boa e nem o preço*" (Grupo 2) |
| | F20 | "*E aí vem né, a questão financeira, porque são alimentos também que são mais caros, e ai a gente entra naquele ciclo né, 'ah ter uma alimentação saudável acaba sendo mais caro', uma questão que contribuiu muito aqui em casa é que minha família é pequena, sou filha única, não tem uma quantidade de pessoas aqui, quando aumenta a quantidade de pessoas na residência aí acaba complicando a questão, mas é isso, é optar por feiras livres mesmo, não pela questão de comprar alimentos in natura no supermercado que a gente sabe que ali tem uma presença maior de agroquímicos*" (Grupo 1) |

(continua)

**Quadro 2 (continuação)**

| SÍNTESE DOS RESULTADOS | | |
|---|---|---|
| **DIMENSÕES** | **CÓDIGO** | **RESPOSTA** |
| Presença de propagandas e promoções | F21 | *"Propaganda de alimento saudável não, não existe. É... a maior parte da propaganda se concentra em folder de mercado, desses pequenos mercados que eu comentei, é... tem também o WhatsApp e tal presente de... é... padarias, outros pequenos comércios e tal que vão, é... contém seu contato né no banco do produto, é... tem cartazes dentro dessas quitandas, desses pequenos comércios, então, a gente tem um, não assédio, mas a gente tem um ambiente que não é legal porque vamos supor que a gente pega uma pessoa que tem a mente cansada da jornada de trabalho, quando ela entra na comunidade, ou no bairro, né, já tem ali salgado R$2,00, é... espetinho não sei quanto, então a chance dela parar ali e comer uma parada assim é grande"* (Grupo 2) |
| Utilização de serviços de entrega de alimentos | F22 | *"Então tem uns combos assim gigantes que dá às vezes pra uma família inteira comer, contando com cunhados, enfim, que forma aquele grupo grande que vem até numa pizza que vem batata-frita, nuggets, filezinho de frango frito né, que é aquelas coisinhas, e um outro negócio, então assim vem um negócio recheado, bastante gordura, bastante coisa bem não saudável, e... aí o combo é R$ 30,00, R$ 40,00, então todo mundo divide e come aquilo dali, que fica um pouco mais em conta e dá pra todo mundo divertir naquele momento, como se fosse uma diversão de final de semana"* (Grupo 1) |
| Presença de hortas comunitárias ou vizinhos com hortas que vendiam ou doavam alimentos | F23 | *"Então assim o poder paralelo tem certo domínio sobre essa parte, então eles não deixam os militantes chegar próximo nem fazer os movimentos com a horta, mas o que a gente via é que ela funcionava assim muito bem, só não era muito de acesso pra qualquer pessoa não, era um pouco restrito, então acho que era por isso inclusive que a gente não podia chegar perto, mas quando eu passava assim pertinho, assim só pra dar uma olhada de curiosidade só pra ver, encontrar alguém ali mexendo pra poder conversar e tentar fazer uma ponte com Manguinhos solidário que era do próprio território e tal ai sempre os avisos era sempre cara deixa isso quieto, não mexe, fica tranquilo ai de boinha, "pô mas saiu no jornal", não, fica tranquilo, deixa isso quieto, deixa isso pra lá, depois a gente conversa melhor sobre, mas aí era muito isso, tinha até girassol plantado no lugar, uma coisa grande, entendeu? Bonita"* (Grupo 1) |
| Recebimento de doações | F24 | *"A gente tem muita gente da Venezuela que veio morar no bairro, então a gente recorre às ONGs né, às igrejas, doações pra gente tá distribuindo pra essas pessoas"* (Grupo 2) |

estratégias para equilibrar custos e benefícios no momento das compras, resultando em maior compra em supermercados, que oferecem condições mais baratas, principalmente após o aumento dos preços dos alimentos devido à pandemia de COVID-19 (Quadro 2; código F18). No entanto, eles tendem a comprar alimentos em pequenos comércios da região, baseando suas escolhas em valores e ideais, quando possível.

Os participantes relataram dificuldade em comprar frutas e verduras, principalmente devido ao alto preço, falta de informação e pela baixa qualidade e variedade dos alimentos encontrados. Também foi relatada a inacessibilidade aos alimentos orgânicos por questões financeiras e baixo acesso físico (Quadro 2; códigos F18 a F20).

Em relação à promoção e divulgação de alimentos, os participantes relataram que as promoções às quais eles têm acesso são mais associadas aos alimentos ultraprocessados e industrializados, além de alimentos vencidos ou próximos do vencimento (Quadro 2; código F21). Também foram relatados exemplos como o "carro do ovo", representando vendedores ambulantes que passam nas ruas oferecendo ovos por um preço abaixo do aplicado em comércios formais ou em promoções de supermercados via folhetos.

Quanto à realização de refeições fora de casa, foi observado que é uma prática que ocorre raramente entre os participantes, sendo realizada somente quando há condições financeiras. E quando se realiza, os locais preferidos são bares, restaurantes e lanchonetes da cidade para consumir sanduíches, sorvetes, pizzas e *yakisobas*.

A aquisição de alimentos prontos por meio de *sites* e aplicativos de entregas ocorre mais frequentemente aos finais de semana ou no trabalho. Há preferência por alimentos considerados não saudáveis, e existe uma preocupação em priorizar lojas de alimentos da comunidade que oferecem o serviço de entrega (Quadro 2; código F22).

Em relação à produção de alimentos, os participantes não têm horta em casa, mas existe ou existiu horta comunitária em boa parte das comunidades. Entretanto, esse espaço não é acessado com frequência pelos moradores. A ausência do poder público dificulta a manutenção das hortas comunitárias, gerando um acúmulo de desvantagens que dificulta o acesso aos alimentos produzidos nesses locais (Quadro 2; código F23).

No bloco de perguntas sobre doações de alimentos, os participantes afirmaram receber doações, principalmente de igrejas, movimentos sociais e organizações não governamentais, além de locais de trabalho e da família (Quadro 2; código F24). Durante a pandemia de COVID-19, os participantes também receberam doações do governo, especialmente quando tinham crianças em idade escolar.

## Discussão

Este é o primeiro estudo que avaliou, de forma qualitativa, a percepção dos moradores de favelas sobre o ambiente alimentar, explorando o acesso aos alimentos em sua vizinhança. A partir da análise dos discursos nos grupos focais, foi construída uma rede temática, cujo principal resultado foi que o acesso aos alimentos é permeado pela falta de informação sobre alimentação, pela renda insuficiente e pela baixa disponibilidade de estabelecimentos que comercializam alimentos saudáveis a preços acessíveis em suas vizinhanças. Dessa forma, as cinco dimensões da alimentação foram identificadas: do direito humano, biológica, sociocultural, econômica e ambiental. E três dimensões principais do conceito de acesso aos alimentos no ambiente alimentar surgiram em suas falas: acesso físico, acesso financeiro e aceitabilidade (cultura e hábitos). A partir dessas dimensões, será realizada a discussão dos resultados encontrados neste estudo.

As favelas geralmente são caracterizadas por um padrão urbanístico irregular, pela carência de serviços públicos essenciais e pela localização em áreas com restrições à ocupação [9]. Em 2021, o Instituto Locomotiva, em parceria com o Instituto Data Favela e a Central Única das Favelas (CUFA), lançou os resultados de uma pesquisa sobre a caracterização de pessoas que moram em favelas, que são cerca de 17,1 milhões no Brasil, representando 8% da população do país [23]. Nas favelas, as iniquidades da sociedade estão presentes no âmbito da oferta de serviços de saneamento básico, saúde e educação e isso se reflete no ambiente alimentar e, consequentemente, no acesso aos alimentos [24,25].

O acesso físico e econômico a alimentos saudáveis e seguros, em quantidade e qualidade adequadas e suficientes, é um direito humano garantido na *Constituição Federal* por meio da *Emenda Constitucional nº 64/2010* [26]. Entretanto, os moradores de favelas se veem frequentemente em situações de acesso escasso aos alimentos saudáveis, como relatado neste estudo, sendo obrigados a recorrer a opções menos saudáveis e mais baratas que estão disponíveis no ambiente.

Dados da pesquisa realizada pelo Instituto Data Favela para verificar os impactos da pandemia da COVID-19 entre as pessoas que moram em favelas, mostrou que 68% dos entrevistados não tiveram dinheiro para comprar comida em pelo menos um dia nas duas semanas anteriores ao levantamento [27]. E a inclusão de alimentos saudáveis na alimentação de pessoas com baixa renda comprometem ainda mais a porcentagem da renda familiar que é destinada à alimentação [28], principalmente em um cenário de inflação dos preços dos alimentos [29] e baixa disponibilidade de alimentos saudáveis de qualidade e com preços acessíveis em regiões vulneráveis [5,6,7,30,31].

Nesse sentido, é importante ressaltar a percepção dos participantes quanto ao que é ou não um alimento saudável, tendo sido frequentemente mencionados os teores de sal, açúcar, micronutrientes, proteína e carboidratos. Essa associação é muito comum do ponto de vista do nutricionismo, que reduz o alimento à sua composição para categorizá-lo entre saudáveis ou não de acordo com a presença ou ausência de alguns nutrientes [32], caracterização perpetuada por muitos anos no Brasil com a utilização da pirâmide alimentar, mencionada por um dos participantes. Além de não ser efetiva, essa classificação acaba por enfraquecer aspectos culturais, sociais e políticos que estão envolvidos com os alimentos e com o ato de se alimentar [33]. O *Guia Alimentar para a População Brasileira* [2] preconiza uma alimentação saudável como sendo aquela que deriva de um sistema alimentar socialmente e ambientalmente sustentável, levando em consideração diversas dimensões da alimentação e a sua complexa relação com a saúde e o bem-estar dos indivíduos.

Dessa forma, embora a ingestão adequada de nutrientes seja essencial para a saúde, outros parâmetros devem ser considerados para caracterizar uma alimentação adequada e saudável, incluindo, entre outros aspectos, as formas de preparo e processamento dos alimentos e as dimensões sociais e culturais das práticas alimentares [34]. Com isso, é importante que a definição de alimentação saudável também inclua o conjunto de fatores que respeitem a identidade e cultura local [2].

Nesse contexto, uma alimentação saudável deve assumir a extensão e o propósito do processamento dos alimentos que compõem a alimentação, e não apenas o seu conteúdo nutricional, considerando saudável uma alimentação baseada em alimentos *in natura* e minimamente processados em detrimento dos alimentos ultraprocessados, como recomenda o *Guia Alimentar para a População Brasileira* [2]. Porém, fazer dos alimentos *in natura* e minimamente processados a base da alimentação vem se tornando cada vez mais complexo devido a fatores como a falta de informação sobre os alimentos e suas classificações, a falta de tempo para o preparo e a dificuldade de acesso aos alimentos saudáveis [2].

Neste estudo, os relatos mostraram diversas barreiras enfrentadas pelas pessoas que residem em favelas para acessar e consumir os alimentos. Além da falta de acessibilidade física e financeira aos alimentos saudáveis, os moradores dessas regiões precisam lidar com: a grande disponibilidade de alimentos ultraprocessados no ambiente, como citado pelos participantes dos grupos focais e evidenciado em diversos estudos brasileiros [35,36,37,38,39,40]; a violência em suas vizinhanças; as muitas propagandas sobre alimentos não saudáveis, resultando em uma confusão no momento de escolher entre as opções [41]; e o tempo despendido com o transporte entre a casa e o trabalho ou local de estudo, que resulta na falta de tempo para comprar e preparar alimentos, visto que gastam mais tempo deslocando-se para esses locais [42,43].

Sendo assim, para promover a alimentação adequada e saudável para a população, são necessários programas e políticas que: incentivem a abertura de estabelecimentos que comercializem alimentos *in natura* e minimamente processados com preços acessíveis; auxiliem na construção e manutenção de hortas comunitárias em áreas de favelas, a fim de reduzir as iniquidades de acesso físico e econômico aos alimentos saudáveis presentes nesses territórios, contribuindo para o aumento do consumo desses alimentos [44]; e promovam políticas de proteção social e urbanização das áreas de favelas, objetivando reduzir a violência e as taxas de homicídios [45]. Vale destacar que as hortas podem promover a conscientização sobre alimentos saudáveis, fortalecer a resiliência da comunidade e contribuir para uma dieta mais diversificada.

Ademais, essas ações de melhoria da infraestrutura urbana podem estimular a abertura de estabelecimentos de comércio de alimentos por transformar espaços outrora precários ou pouco atrativos para esse mercado [3,4]. Por outro lado, o poder público pode incentivar os moradores das comunidades a abrirem esses estabelecimentos de venda de alimentos saudáveis, estimulando e fortalecendo a economia local e auxiliando no seu desenvolvimento. Uma pesquisa do Instituto Data Favela constatou que cerca de 40% dos moradores das favelas do Rio de Janeiro têm seu próprio negócio e 22% pretendem abrir um empreendimento [46]. Outras estratégias inovadoras podem incluir o estímulo à instalação de pontos de venda de alimentos nas proximidades de estações e centros de transporte público, assim como a promoção de iniciativas móveis para a comercialização de alimentos saudáveis. Isso poderia ser realizado por meio da implantação de ônibus urbanos que percorram a cidade oferecendo alimentos *in natura* e minimamente processados ou por meio de vendedores ambulantes que disponham de carrinhos para essa finalidade.

Outra estratégia importante e abrangente consiste na isenção de impostos sobre os alimentos *in natura* e minimamente processados. No Brasil, vivemos em um momento oportuno para debater a tributação, devido às discussões em curso sobre a reforma tributária. Diversas organizações sem fins lucrativos brasileiras, como o Instituto de Defesa do Consumidor (IDEC) e a Aliança de Controle do Tabagismo (ACT) Promoção da Saúde, lideram um movimento em prol de uma reforma tributária saudável, sustentável e solidária (3S). Essa proposta tem como objetivo criar um sistema tributário mais equitativo em relação à renda e ao patrimônio, implementando impostos seletivos sobre produtos prejudiciais à saúde, aumentando as taxas para setores que lucram à custa da saúde e do meio ambiente. A proposta inclui a isenção de impostos para produtos da cesta básica, bem como a introdução de incentivos fiscais para ampliar a oferta e a acessibilidade de alimentos saudáveis para a população [47].

Também são necessárias ações que abordem as barreiras enfrentadas por moradores de favelas para acessar os alimentos saudáveis, como a garantia de transporte público para essas áreas com horários que atendam a população, políticas públicas voltadas para a segurança e combate à violência, ações articuladas entre a saúde e a educação para levar informações sobre a alimentação saudável e prevenção e tratamento de doenças, regulamentação das ações de marketing de alimentos não saudáveis, entre outras medidas que incorporem a complexidade dos problemas relacionados à vida urbana nas favelas.

O estudo apresenta algumas limitações. Primeiro, como os participantes foram informados sobre o contexto do estudo, podem ter dado respostas socialmente desejáveis. Além disso, não foi possível coletar dados não verbais, devido à utilização de recursos *online*. Por fim, a realização do grupo focal de forma virtual pode ter excluído possíveis participantes que não tinham acesso à internet ou que têm dificuldades com o manuseio de ferramentas digitais.

Embora tenha suas limitações, a realização do grupo de forma virtual também é particularmente viável por possibilitar a participação de moradores de favelas de todas as regiões do Brasil, que vivenciam diferentes contextos. Além disso, a condução dos grupos por um pesquisador experiente e treinado em técnicas qualitativas de pesquisa minimiza possíveis interferências dos moderadores sobre os resultados. Também é importante destacar o pioneirismo deste estudo, ao investigar a percepção dos moradores de favelas sobre o ambiente alimentar de sua vizinhança e como ocorre o acesso aos alimentos nesses locais, do ponto de vista das pessoas que nele residem.

## Conclusão

Este estudo representa um avanço ao explorar qualitativamente a percepção dos moradores de favelas em relação ao ambiente alimentar, avaliando o acesso aos alimentos em suas vizinhanças. A análise dos discursos revelou que o acesso dos moradores de favelas a alimentos saudáveis é fortemente influenciado por limitações de recursos fundamentais para uma alimentação saudável, estabelecendo as barreiras: falta de informação sobre alimentos e alimentação; restrição de renda; e escasso acesso a estabelecimentos que oferecem alimentos saudáveis a preços acessíveis. O estudo aponta a necessidade de políticas e programas que incentivem a diminuição dos preços de alimentos, bem como estratégias para melhorar o acesso físico e econômico a esses alimentos nas comunidades. A criação e manutenção de hortas comunitárias, incentivo ao comércio local, concessão de benefícios fiscais aos alimentos *in natura* e minimamente processados, e políticas de proteção social e urbanização das áreas das favelas são apontadas como medidas efetivas para reduzir as iniquidades no acesso aos alimentos saudáveis.

## Colaboradores

L. L. Rocha contribuiu com a concepção e metodologia do estudo, análise dos dados, redação e revisão; e aprovou a versão final. A. A. L. Friche contribuiu com a metodologia do estudo, redação e revisão; e aprovou a versão final. M. Z. Jardim contribuiu com a análise dos dados, redação e revisão; e aprovou a versão final. P. C. P. Castro Junior contribuiu com a redação e revisão; e aprovou a versão final. E. P. Oliveira contribuiu com a redação e revisão; e aprovou a versão final. L. O. Cardoso contribuiu com a redação e revisão; e aprovou a versão final. L. L. Mendes contribuiu com a concepção e metodologia do estudo, redação e revisão; e aprovou a versão final.

## Informações adicionais

ORCID: Luana Lara Rocha (0000-0002-5963-6033); Amélia Augusta de Lima Friche (0000-0002-2463-0539); Mariana Zogbi Jardim (0000-0003-3740-4183); Paulo César Pereira de Castro Junior (0000-0002-6629-9610); Emanuelly Porto Oliveira (0009-0005-9387-9951); Letícia de Oliveira Cardoso (0000-0003-1312-1808); Larissa Loures Mendes (0000-0003-0776-6845).

## Agradecimentos

À Fundação de Amparo à Pesquisa do Estado de Minas Gerais (APQ-01481-21).

## Abstract

*Food availability in the territory can influence food consumption by the population. However, it is important to understand how people perceive their food environment to see how food availability affects consumption in different contexts. This study aimed to assess the perception of the food environment by Brazilian slum residents in their neighborhoods. This is a qualitative study, with online focus groups guided by a script in order to gather collective discourses about access to food in Brazilian slums. The invitation to participate in this study was made through social media, and community leaders and nongovernmental organizations with actions in slums were contacted using the snowball sampling technique. Grounded theory analysis was applied with the technique of thematic networks. Access to food for slum residents involves lack of resources and essential elements for an adequate and healthy diet, such as lack of information about food, low income, and low availability of stores that sell healthy food at affordable prices. Public programs and policies are required to encourage the expansion of food and nutritional security resources, such as vegetable gardens and markets, to increase the supply and sell healthy food at affordable prices in slums. Actions are also required to address the complexity of obstacles faced by slum residents in the access to healthy foods.*

*Perception; Access to Healthy Foods; Slums*

## Resumen

*La disponibilidad de venta de alimentos en el territorio puede influir en el consumo alimentario de la población. Sin embargo, es importante comprender la manera en que las personas perciben su entorno alimentario para comprender cómo esta disponibilidad afecta su consumo en diferentes contextos. El objetivo fue evaluar la percepción de los residentes de favelas brasileñas sobre el entorno alimentario en sus vecindarios. Estudio cualitativo, en el que se formaron grupos focales en línea orientados por un guion con el objetivo de reunir discursos colectivos sobre el acceso a los alimentos en favelas brasileñas. La invitación se dio por medio de las redes sociales y mediante el contacto con líderes comunitarios y organizaciones no gubernamentales que trabajan en favelas, utilizando la técnica de muestreo "bola de nieve". Para el análisis, se utilizó el enfoque de la* grounded theory *(teoría fundamentada) y, como técnica, se empleó el análisis de redes temáticas. El acceso a los alimentos de los habitantes de las favelas está permeado por la falta de recursos y elementos fundamentales para una alimentación adecuada y sana, tales como: la falta de información sobre la alimentación, los bajos ingresos y la poca disponibilidad de establecimientos que vendan alimentos sanos a precios asequibles. Se necesitan programas y políticas públicas para fomentar la ampliación de equipos de seguridad alimentaria y nutricional, como huertas y mercadillos, que aumenten la oferta y vendan alimentos sanos a precios asequibles en las favelas. También se necesitan acciones para abordar la complejidad de las barreras que enfrentan los residentes de las favelas para acceder a alimentos sanos.*

*Percepción; Acceso a Alimentos Saludables; Favelas*

Recebido em 11/Jul/2023
Versão final reapresentada em 30/Nov/2023
Aprovado em 07/Dez/2023
